# Insecticide-treated bed nets (ITN) ownership and utilization patterns among caregivers with children under five years: A community-based cross-sectional study in Battor, North Tongu District, Ghana

**DOI:** 10.1371/journal.pgph.0004228

**Published:** 2025-02-06

**Authors:** Emmanuel Abu Bonsra, Petra Amankwah Osei, Akua Grace Sekyi, Gideon Amankwah Kyere

**Affiliations:** 1 Department of Population and Behavioural Sciences, Fred N. Binka School of Public Health, University of Health and Allied Sciences, Hohoe, Ghana; 2 Family and Community Health, Fred N. Binka School of Public Health, University of Health and Allied Sciences, Hohoe, Ghana; PLOS: Public Library of Science, UNITED STATES OF AMERICA

## Abstract

Insecticide-treated nets (ITNs) are essential for malaria prevention, significantly reducing the incidence of malaria, particularly among children under five. However, the utilization of ITNs varies widely due to several factors. This study aimed to assess ITN ownership and utilization patterns among caregivers of children under five years old. A cross-sectional design was employed, utilizing a multi-stage sampling to collect quantitative data from 211 caregivers. Data analysis was performed using descriptive and inferential statistics via Stata 17. Of the total respondents (211), 77 (36.5%) own ITNs, while 134 (63.5%) do not own ITNs. Chi-square analysis revealed significant associations between ITN knowledge and factors such as sex (p = 0.025), educational level (p = 0.043), marital status (p < 0.001), religion (p = 0.020), challenges faced in using ITNs (p = 0.012), affordability (p = 0.001), and cultural beliefs (p = 0.003). Binary regression analysis indicated that caregivers facing challenges in using ITNs were 50% less likely to utilize them compared to those without such challenges (aOR = 0.50, 95% CI = 0.26–0.96, p = 0.040). Additionally, caregivers who found ITNs inconvenient or hot were 30% less likely to use them compared to those who viewed them as costly (aOR = 0.70, 95% CI = 0.43–1.33). To enhance ITN utilization, the Ghana Health Service should tailor interventions to address practical barriers and improve educational outreach, bridging the gap between knowledge and usage.

## Introduction

### Background

Malaria remains a persistent global health challenge, particularly in regions with limited access to healthcare and resources [1]. Malaria is a complicated illness with widely varying epidemiology and clinical presentations that continues to be the world’s largest cause of mortality [2]. Malaria diagnosis has traditionally been focused mostly on signs and symptoms [2]. Thus, malaria was generally treated presumptively, resulting in misuse of anti-malarial medications, inefficiency of resources, and avoidable suffering, particularly if fever was coupled with other diseases [3]. Among the most susceptible groups affected by this parasitic infection are children under the age of five [3]. The devastating impact of malaria on this demographic is evident in its high morbidity and mortality rates, posing a significant public health concern worldwide [4]. A child dies every minute from malaria in Africa where it is estimated that 9 out of 10 malaria deaths occur [5]. Furthermore, some studies have noted that many public basic healthcare institutions lacked essential diagnostic tools, such as microscopes, which hinder effective malaria diagnosis and treatment [6].

In 2022, nearly half of the world’s population was at risk of malaria [7]. While sub-Saharan Africa carries a disproportionately high share of the global malaria burden, the WHO regions of South-East Asia, Eastern Mediterranean, Western Pacific, and the Americas also report significant numbers of cases and deaths [7]. There were an estimated 249 million cases of malaria in 2022, and the estimated number of malaria deaths stood at 608 000. In 2022, the African Region was home to 94% and 95% of malaria cases and deaths, respectively [7]. Children under 5 years of age are the most vulnerable group affected by malaria; in 2022, they accounted for nearly 80% of all malaria deaths in the WHO African Region [7].

Globally, the prevalence of malaria has shown a gradual decline over the years, but it continues to be a major public health challenge [8]. According to the World Health Organization, in 2020, there were approximately 241 million cases of malaria reported [9], most malaria cases and deaths indeed occurred in sub-Saharan Africa, accounting for about 95% of all malaria cases and deaths, which aligns with your assertion that sub-Saharan Africa bears the brunt of the burden [10]. Children under five years old accounted for approximately 77% of all malaria deaths globally [10,11].

In combating the spread of malaria, insecticide-treated bed nets (ITNs) have emerged as a crucial intervention [12]. Insecticide-treated bed nets are recommended for use by the World Health Organization (WHO) and have been implemented in both high-income and low- and middle-income countries (LMICs) [13]. These efforts are integral to achieving the Sustainable Development Goals (SDGs) target of ending malaria epidemics by 2030 [14]; and [12]. However, despite these initiatives, challenges persist in ensuring universal access and consistent utilization of ITNs, especially among caregivers with children under five years old [15]. In developed countries, ITNs are primarily used by travellers to malaria-endemic areas to prevent malaria transmission [15]. In low- and middle-income countries (LMICs), insecticide-treated bed nets are distributed through mass distribution campaigns and are primarily used by children under five years of age and pregnant women [16]. These nets act as a physical barrier, effectively preventing mosquito bites during sleep and subsequently reducing the risk of malaria transmission [17]. Their effectiveness in reducing malaria cases, particularly among vulnerable populations such as children under five, has been well-documented in numerous studies and public health initiatives [18].

However, several contextual factors intricately influence ITN utilization among caregivers with children under five [19] Socio-economic disparities, varying cultural beliefs, geographical accessibility to healthcare facilities, educational backgrounds, and past exposure to health interventions significantly impact the adoption and sustained use of ITNs within communities [20]. Emerging challenges such as resistance to insecticides, issues related to ITN durability, and behavioural factors affecting sustained utilization highlight the need for updated and context-specific data [20].

In Ghana, malaria remains a significant public health challenge, with an estimated 5.5 million cases and 16,000 deaths in 2019 [21]. Insecticide-treated bed nets have also been implemented as part of the national malaria prevention strategy in Ghana [22]. According to the data from the 2019 Ghana Malaria Indicator Survey, 74% of households owned at least one insecticide-treated bed nets [23]. However, the study indicated that the usage rate for children under five years was only 54%, the reported utilization of insecticide-treated bed nets among children under five was found to be approximately 61.88% [23]. Therefore, this study sought to determine the insecticide-treated bed nets (ITN) ownership and utilization patterns among caregivers with children under five years.

## Materials and methods

### Study design and data source

In this study, a cross-sectional design was adopted to investigate the prevalence and determinants of insecticide-treated bed net (ITN) ownership and utilization patterns among caregivers of children under five years old in Ghana. This design allowed for a comprehensive examination of insecticide-treated bed net ownership and associated factors within this demographic [15]. The rationale for selecting a cross-sectional design was its ability to capture a snapshot of attitudes and concerns regarding insecticide-treated bed net ownership and utilization at a specific point in time. Structured questionnaires were employed to gather data from a representative sample of caregivers, focusing on insecticide-treated bed net ownership, knowledge of usage, barriers to consistent utilization, and demographic characteristics. The study aimed to collect numerical data on ownership rates and factors influencing insecticide-treated bed net utilization. Statistical analysis methods, including regression, were used to identify relationships between various factors (such as demographics and socio-economic status) and insecticide-treated bed net patterns. By employing this quantitative approach, the study sought to provide insights into insecticide-treated bed net ownership prevalence among caregivers of young children and analyse factors associated with utilization. Adjustments were made based on available resources and specific research objectives.

### Study site description

North Tongu District is in the Volta Region of Ghana and has a rich history and geographical significance, with Battor Dugame as its capital [21]. The district lies within the Tropical Savannah Grassland zone, with the Volta River cutting through it from north to south. According to the 2010 Population and Housing Census, North Tongu District had a population of approximately 89,777, with a higher proportion of females (52.7%) compared to males (47.3%) [22]. The area is predominantly rural, with about 60% of its population living in rural settings. The district has a total of 20 operational health facilities, which include one district hospital, several health centers, and Community-Based Health Planning and Services (CHPS) compounds. Malaria remains a significant public health concern in the district, despite ongoing malaria control programs, including insecticide-treated bed nets (ITNs) and indoor spraying [21]. These efforts have led to some improvements in malaria prevention, but the disease continues to affect many residents, particularly in rural areas, underscoring the need for continued prevention strategies and healthcare.

### Study population

The target population consisted of caregivers (both women and men) with children under five in Battor, located in the North Tongu District of Ghana. All caregivers actively providing care for at least one child under the age of five years and available during the data collection period were included in this study. Individuals who did not have primary caregiving responsibilities for children under five, as well as caregivers who were unreachable or unavailable during the data collection period, were excluded from the study.

### Sampling strategy

This study investigates insecticide-treated bed net (ITN) ownership and utilization patterns among caregivers of children under five years in Battor, North Tongu District, Ghana. To ensure a representative sample, a multi-stage sampling method was applied, which included three stages of selection. In the first stage, 10 communities were randomly selected from a list provided by local health authorities, ensuring a mix of urban, peri-urban, and rural settings within the district. This approach helped to capture the diversity of socio-economic conditions across the region. In the second stage, households with children under five years of age were identified in each of the selected communities. A list of eligible households was compiled with assistance from local health workers or health facilities. Systematic sampling was then used to select households by choosing every fifth household, ensuring that the sample size remained manageable while being representative of various household types. In the third stage, caregivers of children under five years were identified within the selected households. If there were multiple eligible caregivers in a household, one caregiver was randomly selected using a lottery system. This random selection ensured an unbiased sample of participants. The final sample consisted of 211 caregivers, providing a diverse and representative group. Despite logistical constraints and time limitations, absentees were not replaced, and no second visits were conducted. This decision was made to maintain the feasibility of the study while keeping the sample representative of the district. The random sampling method ensured that the diversity of the sample was preserved, even with these limitations. The three-stage sampling approach allowed for comprehensive data collection on ITN ownership and utilization patterns, reflecting the wider socio-economic conditions of the North Tongu District.

### Sample size determination

The formula utilized will be:


$$n=\frac{z^{2}\times p\left(1-p\right)}{d^{2}}$$

Where:

n = Sample size.

Z = The z-score that corresponds with a 95% confidence level, typically 1.96.

P = Estimated prevalence of ITN bed ownership (set at 50% due to the lack of available data).

*d* = Margin of error set at 5% (0.05).

Nonresponsive rate 10%

The calculation resulted in a sample size of 201 caregivers. To account for non-response and incomplete data, 10% was added, leading to a final sample size of 211 participants.

This estimation assumes an ITN ownership rate of 50%, which provides the maximum sample size in the absence of prior district-specific prevalence data. Although studies in other parts of Ghana (e.g., sources [24]) report ITN ownership rates that vary, the estimated rate of 50% ensures that the sample size is adequate for a conservative analysis. The target population comprised caregivers of children under five years old in the North Tongu District, a subpopulation distinct from the total district population of approximately 90,000. The multi-stage sampling strategy used in this study ensured a diverse and representative sample, suitable for analyzing ITN ownership and utilization patterns among this specific group.

### Data collection methods and instruments

To gather socio-demographic and pertinent information regarding ITN ownership and utilization among caregivers with children under five in Ghana, in-person interviews conducted by trained interviewers were utilized. A structured questionnaire tailored specifically for the study’s objectives served as the primary tool for data collection. The questionnaire encompassed factors influencing ITN acceptance, reasons for non-utilization, and demographic details relevant to caregivers. Insights from existing literature and pre-testing for clarity and relevance informed the development of the questionnaire, which aimed to capture nuanced insights into ITN ownership and utilization patterns. It offered a comprehensive range of response options to gather detailed data on caregivers’ perspectives. Interviewers were recruited specifically for this study and underwent extensive training in questionnaire administration and ethical considerations. The recruitment period for this study, from December 10, 2023, to August 10, 2024, was extended due to logistical and environmental challenges. A significant factor was the overflow of water from the Akosombo Dam (Akosombo Dam Spillage), which caused widespread displacement of residents in the Mepe and Battor areas of the North Tongu District.

### Ethics approval and consent to participate

The study received approval from the University of Health and Allied Sciences Research Ethics Committee (UHAS-REC) under reference number UHAS-REC B.20 [032]23-24. Prior to administering the questionnaires, permission was obtained from the North Tongu District Assembly. All interviews were conducted with the full consent of participants. For participants under 18 years of age, written informed consent was obtained from their parents or guardians. In addition, verbal assent was sought from the minors to ensure they understood the purpose of the study and agreed to participate. This dual consent process (from both parents/guardians and minors) was approved by UHAS-REC to ensure ethical compliance in the involvement of child participants. For all other participants, either written or thumb-printed informed consent was obtained. Verbal consent was also sought to ensure full understanding, especially for individuals who required further clarification or preferred verbal communication. This process was sanctioned by UHAS-REC. The recruitment period for this study started on 10th December 2023 and ended on August 10th, 2024. Confidentiality was maintained by using pseudonyms, and access to the encrypted, password-protected database was restricted to the Principal Investigator and research supervisor.

### Study variables

#### Outcome variables.

The primary outcome variables in this study were the ownership and utilization rates of ITNs.

### Prevalence variables

In the data analysis, a composite variable was created to assess ITN ownership and utilization patterns among the respondents. The criteria for creating this composite variable included whether the respondent currently owns ITNs for malaria prevention (Yes/No), the number of ITNs owned (None/One/Two/Three, and Four or more), and whether the respondent and their child under five slept under an ITN the night before the survey (Yes/No). ITNs. ITN ownership and utilization were further analysed using quartiles. “Low ownership/utilization” refers to respondents in the lower quartiles, while “High ownership/utilization” refers to those in the upper quartiles. This categorization allows for a more granular analysis of ownership and usage patterns, highlighting differences in ITN access and use among the respondents. Ownership was determined by the number of ITNs possessed, while utilization was based on recent usage by the caregiver and their child. This method allowed for a more granular categorization of the number of ITNs owned, with the categories including None, One, Two, Three, and Four or more ITNs, providing a clearer distinction of ITN ownership levels.

### Knowledge variables

Knowledge of insecticide-treated nets (ITNs) among caregivers with children under five was assessed using the following variables, heard of malaria, causes of malaria, and ITN usage guidelines. A composite variable was generated, and caregivers were classified into high or low knowledge groups based on the 75th percentile threshold of correct responses.

#### Explanatory variables.

The explanatory variables included socio-demographic factors (such as age, gender, education level, income, and marital status), knowledge-related, and access factors served as explanatory variables influencing these outcomes.

### Statistical analysis

Data collected from participants was entered into the Kobo Collect application and exported into STATA v17.0 for analysis. To ensure the quality of the data entered, the data was double-checked to address discrepancies during data entry. All numerical data was analysed using descriptive statistics. To establish the associations between variables, Chi-square and logistic regression p-values of less than 0.05 were considered statistically significant at a 95 percent confidence interval. Tables were used to present the results.

## Result

### Socio-demographic characteristics

Table 1 shows the socio-demographic characteristics of the participants. Out of the 211 participants the majority are female (81%) with a mean age of approximately 20 years (SD = 0.82). Most participants belong to the Akan ethnic group (19.0%), followed by Ewe (50.2%), Mole/Dagbani (15.6%), and Ga/Dangme (15.2%). In terms of education, the largest group has completed Junior High School or Middle School (33.6%), while a significant portion of participants are artisans (49.3%) and identify as Christians (74%). Most participants are married (59%), and the majority live in households with 1-5 members (82.9%).

**Table 1:** Socio-demographic characteristics of participants.

Variable	Frequency (N = 211)	Percentage (%)
Mean age X (SD)	X (SD) = 20 (0.82)	
**Age**		
16 – 25	78	37.0
26 – 37	68	32.2
40-max	65	30.8
**Sex**		
Female	171	81.0
Male	40	19.0
**Ethnicity**		
Akan	40	19.0
Ewe	106	50.2
Ga/Dangme	32	15.2
Mole/Dagbani	33	15.6
**Educational**		
JHS/ Middle school	71	33.6
No formal education	12	6.0
Primary	28	13.3
SHS/SSS/O Level/A Level	55	26.1
Tertiary	45	21.3
**Marital status**		
Divorced/Separated	14	7.0
Married	124	59.0
Never married	71	34.1
Widowed	2	0.9
**Employment status**		
Artisan	104	49.3
Civil/ Public servant	55	26.1
Retiree	3	1.4
Unemployed	49	23.2
**Religion**		
Africa Tradition	9	2.3
Christianity	156	74.0
Islam	46	21.8
**Household size**		
1-5	175	82.9
6-max	36	17.1

### Prevalence of insecticide-treated net (ITN) ownership among caregivers

Table 2 shows that 86.1% of the 211 caregivers currently own insecticide-treated nets (ITNs) for malaria prevention, with 43.3% possessing three or more nets. Among those who do not own ITNs, the primary reason is not being present during distribution (76%). Most participants acquired their ITNs from health facilities (55%) or community programs (32%). Additionally, 66.9% of caregivers and their children under five slept under an ITN the night before the survey, and 55% of participants consider using ITNs to be very beneficial for their households.

**Table 2:** Prevalence of insecticide-treated net (ITN) ownership.

Variable	Frequency (N = 211)	Percentage (%)
**Currently own insecticide-treated nets (ITNs) for malaria prevention purposes**		
No	29	13.9
Yes	179	86.1
**If No; reason for not owning insecticide-treated nets (ITNS)**		
No mosquito at my residence	5	17.2
Not around during distribution	22	76.0
Shortage of ITNs	2	7.0
**Number of ITNs currently possess for household**		
None	26	12.3
One	40	19.1
Two	54	25.7
Three and above	91	43.3
**Acquire the insecticide-treated nets (ITNs)**		
Community program	67	32.0
Friend/Relative	26	12.3
Health facility	115	55.0
Pharmacy Shop	3	1.4
**You and your child under five slept in ITN the night before this survey**		
No	72	34.1
Yes	139	66.9
**Beneficial in sleeping in ITNs with your household**		
Beneficial	70	33.2
Not beneficial	13	6.2
Somehow beneficial	12	5.7
Very beneficial	116	55.0

**Fig 1:**
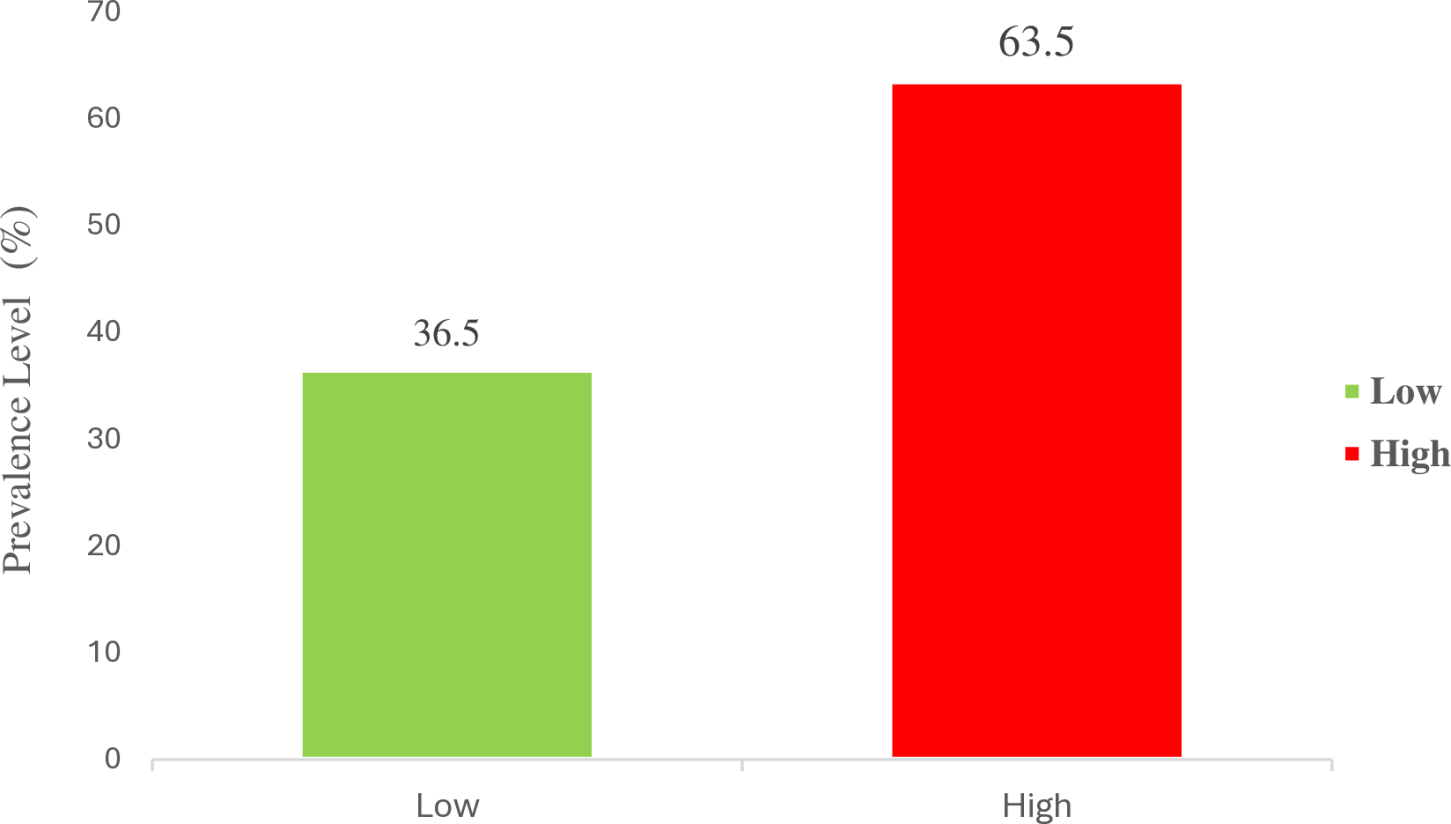
Prevalence of insecticide-treated net (ITN) ownership.

Fig 1 illustrates the overall prevalence of insecticide-treated net (ITN) ownership among the 211 respondents. The respondents were categorized based on their ITN ownership and utilization levels. Of the respondents, 77 (36.5%) own ITNs, while 134 (63.5%) do not own

### Knowledge of ITN usage among caregivers with children under five

Table 3 shows that 97.3% of caregivers have heard of malaria, with 91.5% correctly identifying the bite of an infected mosquito as the cause. Despite this high awareness, 55.1% have not heard of insecticide-treated nets (ITNs). Among those familiar with ITNs, 51% strongly agree, and 46.5% agree that the primary purpose of using ITNs in children under five is to prevent malaria, with similar agreement levels on the need to use ITNs every night. However, 65% disagree that ITNs should be replaced or retreated every two years or after 20 washes, with most information about ITN usage coming from health facilities (60%).

**Table 3:** Knowledge of ITN usage among caregivers with children under five.

Variable	Frequency (n = 211)	Percentage (%)
**Heard of malaria**		
No	5	2.4
Yes	206	97.3
**Causes of Malaria**		
Walking in the sun	4	2.0
Tiredness	4	2.0
Bite of Infected Mosquito	193	91.5
Not eating well	6	3.0
Don’t Know	4	2.0
**Heard of insecticide-treated nets (ITNs)**		
No	113	55.1
Yes	92	44.8
**Primary purpose of using insecticide-treated nets (ITNs) in children under five is to prevent malaria**		
Strongly agree	107	51.0
Agree	98	46.5
Disagree	5	2.4
Strongly disagree	1	0.5
**ITNs should be used every night to effectively prevent malaria in children under five**		
Agree	98	46.5
Disagree	5	2.4
Strongly agree	107	51.1
Strongly disagree	1	
**ITNs should be replaced or retreated with insecticide for effective malaria prevention every 2 years or after 20 washing**		
Disagree	135	65.0
Agree	18	9.0
Strongly agree	56	27.0
**Sources of information acquire about ITN usage for children under five**		
Health facilities	126	60.0
Community programs	43	20.4
Media (TV, radio, etc.)	36	17.1
Family/friends	6	2.8

**Fig 2:**
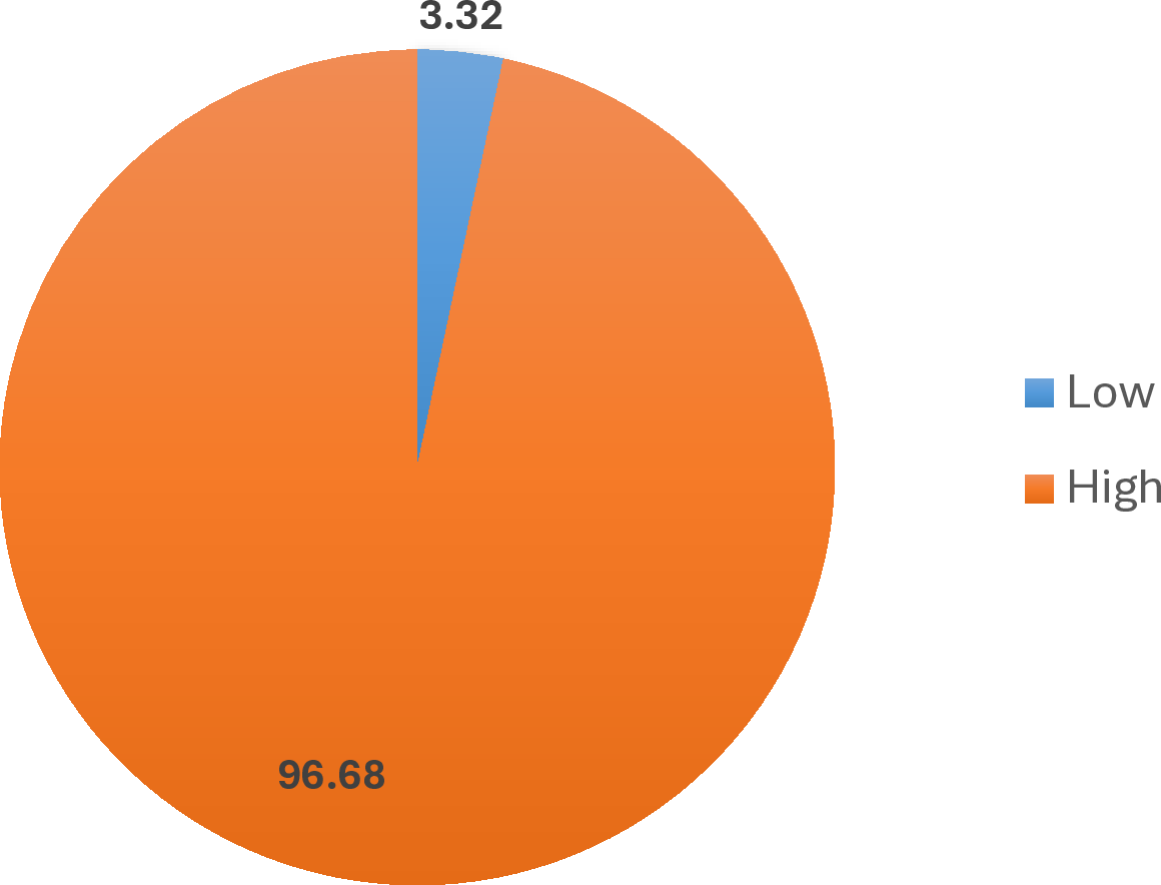
Knowledge of insecticide-treated nets (ITNs).

Fig 2 shows that 96.68% of caregivers fall into the high knowledge category, while only 3.32% are classified as having low knowledge. This indicates that most caregivers are well-informed about the importance and proper use of ITNs for malaria prevention.

### Barriers to the utilization of insecticide-treated nets

Table 4 shows barriers to the Utilization of ITNs. The result shows that 46.5% of caregivers have faced challenges in consistently using insecticide-treated nets (ITNs) for children under five. The primary barrier is inconvenience or heat, cited by 84.3% of those facing challenges, while issues like allergies (9%) and cost (2.3%) are less common. Affordability is not a significant barrier, with 47.4% finding ITNs affordable and 46.9% finding them very affordable. Cultural beliefs do not significantly impact ITN use, as 50.7% strongly disagree and 36.9% disagree that cultural beliefs affect their decision or ability to use ITNs.

**Table 4:** Barriers to the utilization of insecticide-treated nets.

Variable	Frequency (n = 211)	Percentage (%)
**Faced challenges in consistently using ITNs for your children under five**		
No	113	54.5
Yes	98	46.5
**Main challenges faced in using ITNs regularly**		
Allergic to chemical	19	9.0
Availability	5	2.3
Cost	5	2.3
Inconvenience/ heat	178	84.3
Shape texture	4	1.9
**Affordable ITNs for household**		
Affordable	100	47.4
Moderately affordable	9	4.3
Not affordable	3	1.4
Very affordable	99	46.9
**Cultural beliefs significantly affect decision or ability to use ITNs for your children under five**		
Agree	18	8.5
Disagree	78	36.9
Strongly agree	8	3.8
Strongly disagree	107	50.7

### Association between factors influencing the prevalence of insecticide-treated net utilization

Table 5 shows the association between socio-demographic factors, barriers, and knowledge, of utilization of insecticide-treated nets (ITNs) using Chi-square analysis. Sex shows significant association with (p = 0.025), educational level (p = 0.043), marital status (p < 0.001), religion (p = 0.020), challenges faced in using ITNs (p = 0.012), affordability (p = 0.001), and cultural beliefs (p = 0.003) all have significant associations with ITN knowledge.

**Table 5:** Association between factors influencing the prevalence of insecticide-treated net utilization.

Variables	Knowledge level		P-value
	Low level	High level	
**Age**			**0.449**
16 - 25	3 (4.41)	77 (98.7)	
26 - 37	3 (4.41)	65 (95.6)	
40-max	3 (4.62)	62 (95.4)	
**Sex**			**0.025**
Female	3 (1.75)	168 (98.3)	
Male	4 (100)	36 (90.0)	
**Ethnicity**			**0.607**
Akan	5 (4.72)	101 (95.3)	
Ewe	1 (2.5)	39 (97.5)	
Ga/Dangme	0 (0.00)	32 (100.0)	
Mole/Dagbani	3 (3.03)	32 (96.9)	
**Educational**			**0.043**
JHS/ Middle school	3 (4.2)	68 (95.7)	
No formal education	2 (16.6)	10 (83.3)	
Primary	0 (0.00)	28 (100.0)	
SHS/SSS/O Level/A Level	0 (0.00)	55 (100.0)	
Tertiary	2 (4.4)	43 (96.5)	
**Marital status**			**<0.001**
Divorced/Separated	2 (14.3)	12 (85.7)	
Married	2 (1.61)	122 (98.4)	
Never married	2 (2.8)	69 (97.2)	
Widowed	1 (50.0)	1 (50.0)	
**Employment status**			**0.966**
Artisan	3 (2.8)	101 (97.1)	
Civil/ Public servant	2 (3.64)	53 (96.4)	
Retiree	0 (0.00)	3 (100.0)	
Unemployed	2 (4.1)	47 (95.9)	
**Religion**			**0.020**
Africa Tradition	1 (11.1)	8 (88.9)	
Christianity	2 (1.28)	154 (98.7)	
Islam	4 (8.7)	42 (91.3)	
**Household size**			0.843
1-5	6 (3.43)	169 (96.5)	
6-max	1 (2.78)	35 (97.2)	
**Have you faced challenges in consistently using ITNs for your children under five?**			**0.012**
**No**	0 (0.00)	113 (100.0)	
**Yes**	5 (5.43)	87 (94.6)	
**Main challenges faced in using ITNs regularly**			**0.304**
Allergic to chemical	1 (5.3)	18 (94.7)	
Availability	1 (20.0)	4 (80.0)	
Cost	1 (20.0)	4 (80.0)	
Inconvenience/ heat	2 (3.4)	57 (96.6)	
Shape texture	0 (0.00)	4 (100.0)	
**Affordable ITNs for household**			**0.001**
Affordable	1 (1.0)	96 (98.9)	
Moderately affordable	2 (22.2)	7 (77.8)	
Not affordable	0 (0.0)	3 (100)	
Very affordable	2 (2.0)	97 (97.9)	
**Cultural beliefs significantly affect decision or ability to use ITNs for your children under five**			**0.003**
Agree	2 (13.3)	13 (86.6)	
Disagree	2 (2.5)	76 (97.4)	
Strongly agree	1 (12.5)	7 (87.5)	
Strongly disagree	0 (0.0)	107 (100.0)	

### Association between factors influencing the prevalence of insecticide-treated net utilization

Table 6 shows the binary regression relationship between socio-demographic characteristics, barriers, influencing prevalence of the utilization of insecticide-treated nets (ITNs). The result shows that caregivers with Children under Five Years from the Ga/Dangme ethnic group having lower odds of 0.37 times less likely of utilization ITN compared to the Akan ethnicity, as indicated by an adjusted odds ratio (aOR = 0.37, 95% CI = 0.15–0.92, p = 0.033). Caregivers with tertiary education had lower odds of 30% less likely to have a high ITN utilization compared to those with junior high school/middle school education (aOR = 0.31, 95% CI: 0.11–0.92, p = 0.035). Caregivers who face challenges in using ITNs consistently are 50% less likely to utilize them (aOR = 0.50, 95% CI = 0.26–0.96, p = 0.040) compared to those who do not face challenges. ITNs being “very affordable” is associated with lower utilization (cOR = 0.45, 95% CI = 0.25-0.83, p = 0.011), though this association is not significant when adjusted for other factors (aOR = 0.63, 95% CI = 0.31–1.26, p = 0.198). Also, caregiver’s who finds ITN as inconvenience or heat are 30% less likely to use ITN compared to those who see at as costly (aOR= 0.70 95% CI = 0.43–1.33), 0.000)

**Table 6:** Association between factors influencing the prevalence of insecticide-treated net utilization.

Variable	Prevalence of Utilization of ITNs		cOR (95% CI), p-value	aOR (95% CI), p-value
**Variables**	**Low**	**High**		
**Age**				
16 - 25	28 (35.9)	50 (64.1)	**Ref**	**Ref**
26 - 37	2 3 (33.8)	45 (66.2)	2.31 (1.50–3.54), 0.471	1.77 (0.98–3.19), 0.055
40-max	26 (40.0)	39 (60.0)	2.62 (1.52–4.51), 0.721	1.01 (0.43–2.33), 0.975
**Sex**				
Female	60 (35.1)	111 (64.9)	**Ref**	**Ref**
Male	17 (42.5)	23 (57.5)	0.56 (0.38–0.83), **0.004**	0.64 (0.40–1.01), 0.061
**Ethnicity**				
Akan	30 (28.3)	76 (71.7)	Ref	Ref
Ewe	14 (35.0)	26 (65.0)	0.73 (0.33–1.59), 0.432	0.69 (0.28–1.68), 0.416
Ga/Dangme	17 (53.13)	15 (46.8)	**0.34 (0.15**–**0.78), 0.011**	**0.37 (0.15**–**0.92), 0.033**
Mole/Dagbani	16 (48.5)	17 (51.5)	**0.41 (0.18**–**0.93), 0.034**	0.42 (0.17–1.03), 0.061
**Educational**				
JHS/ Middle school	19 (26.7)	52 (73.2)	**Ref**	**Ref**
No formal education	5 (41.6)	7 (58.3)	2.22 (0.95–5.16), 0.062	1.58 (0.75 4.1), 0.356
Primary	7 (25.0)	21 (75.0)	1.62 (0.71–3.65), 0.243	1.78 (0.72–4.41), 0.207
SHS/SSS/O Level/A Level	23 (41.8)	32 (58.2)	1.01 (0.56–1.84), 0.950	1.50 (0.75–3.0), 0.248
Tertiary	23 (51.1)	22 (48.9)	**0.3 (0.15**–**0.76), 0.009**	**0.31 (0.11**–**0.92), 0.035**
**Marital status**				
Divorced/Separated	5 (35.7)	9 (64.3)	Ref	Ref
Married	43 (34.6)	81 (65.3)	**0.08 (0.01**–**0.48), 0.005**	**0.05 (0.00**–**0.9), 0.047**
Never married	27 (38.0)	44 (61.9)	0.18 (0.03–0.53), 0.064	0.04 (0.00–1.25), 0.068
Widowed	2 (100.0)	0 (0.00)	0.928 (0.08–10.70), 0.953	0.15 (0.00–7.03), 0.336
**Employment status**				
Artisan	**33 (31.7)**	**71 (68.3)**	Ref	Ref
Civil/ Public servant	25 (45.5)	30 (54.5)	0.56 (0.28–1.09), 0.089	0.87 (0.34–2.23), 0.787
Retiree	3 (100)	0 (0.00)	0.06 (0.00–1.33), 0.076	
Unemployed	16 (32.6)	33 (67.4)	0.95 (0.46–1.95), 0.892	0.87 (0.38–1.97), 0.743
**Religion**				
Africa Tradition	4 (44.4)	5 (55.6)	Ref	Ref
Christianity	54 (34.6)	102 (65.4)	**9.75 (1.64**–**57.86), 0.312**	1.23 (0.03–49.15), 0.910
Islam	19 (41.3)	27 (58.7)	**13.16 (3.42**–**50.68), 0.591**	1.88 (0.14–24.21), 0.625
**Household size**				
1-5	67 (38.3)	108 (61.7)	Ref	Ref
6-max	10 (27.8)	26 (72.2)	2.99 (0.96–9.2), 0.057	0.43 (0.04–3.83), 0.04s3
**Challenges in consistently using ITNs for your children under five**				
No	30 (26.5)	83 (73.5)	Ref	Ref
Yes	43 (46.7)	49 (53.3)	0.41 (0.22–0.73), 0.003	**0.50 (0.26**–**0.96), 0.040**
**Main challenges faced in using ITNs regularly**				
Allergic to chemical	12 (63.2)	7 (36.8)	**Ref**	**Ref**
Availability	3 (60.0)	2 (40.0)	0.31 (0.50–1.04), **0.000**	0.77 (0.98–3.19), 0.075
Cost	3 (60.0)	2 (40.0)	0.62 (0.52–0.91), **0.571**	1.01 (0.43–2.33), 0.975
Inconvenience/ heat	23 (38.9)	36 (61.0)	**0.91 (0.45**–**0.73), 0.001**	**0.70 (0.43**–**1.33), 0.000**
Shape texture	2 (50.0)	2 (50.0)	0.31 (0.50–1.54), **0.000**	0.77 (0.98–1.19), 0.045
**Affordable ITNs for household**				
Affordable	26 (26.8)	71 (73.2)	Ref	Ref
Moderately affordable	3 (33.3)	6 (66.7)	0.73 (0.17–3.14), 0.675	0.77 (0.15–3.96), 0.758
Not affordable	2 (66.7)	1 (33.3)	0.18 (0.01–2.10), 0.173	0.22 (0.01–3.01), 0.258
Very affordable	44 (44.4)	55 (55.6)	**0.45 (0.25**–**0.83), 0.011**	0.63 (0.31–1.26), 0.198
**Cultural beliefs significantly affect decision or ability to use ITNs for your children under five**				
Agree	4 (26.7)	11 (73.3)	**Ref**	Ref
Disagree	31 (39.7)	47 (60.3)	**0.04 (0.01**–**0.20), 0.000**	0.60 (.03–11.71), 0.739
Strongly agree	4 (50.0)	4 (50.0)	**0.02 (0.001**–**0.51), 0.017**	0.45 (0.01–26.97), 0.704
Strongly disagree	37 (34.6)	70 (65.4)	0.96 (0.15–5.89), 0.966	2.21 (0.109–44.86), 0.604

## Discussion

Insecticide-treated nets (ITNs) are a critical tool in the fight against malaria, a disease that remains a significant public health challenge in many parts of the world. Effective use of ITNs can substantially reduce malaria transmission and its associated health impacts, particularly among vulnerable populations such as children under five years old. Despite the recognized benefits of ITNs, their actual utilization and the factors influencing their use vary widely. The objectives of this research are threefold: first, to assess the prevalence of ITN ownership among caregivers; second, to evaluate the knowledge of ITN usage among these caregivers; and third, to identify the barriers that impact ITN utilization. By addressing these objectives, the research aims to provide insights into how socio-demographic factors, knowledge, and practical challenges influence ITN use, thereby informing strategies to enhance the effectiveness of malaria prevention programs.

This current study found that 86.1% of the 211 caregivers currently own insecticide-treated nets (ITNs) for malaria prevention, with 43.3% possessing three or more nets. Among those who do not own ITNs, the primary reason is not being present during distribution (76%). Most participants acquired their ITNs from health facilities (55%) or community programs (32%). Additionally, 66.9% of caregivers and their children under five slept under an ITN the night before the survey, and 55% of participants consider using ITNs to be very beneficial for their households. Fig 1, illustrates the overall prevalence of insecticide-treated net (ITN) ownership among respondents, categorized as “Low” and “High” prevalence levels, out of the 211 individuals, 77 (36.5%) own ITNs with a low frequency, and 134 (63.5%) own ITNs with a high frequency. The ownership rate of 86.1% in the current study is notably higher than the 44% ownership reported in Guinea [20], where household ITN ownership was significantly influenced by factors such as household size and education level of the household head. This disparity suggests that community-based distribution strategies in Ghana may be more effective than those implemented in Guinea. The current study found that 66.9% of caregivers and their children slept under an ITN the previous night, consistent with findings from a study in Ho Municipality, Ghana, which reported a utilization rate of 66.4% [20]. However, it is lower than the 73.3% utilization reported in Eastern Ethiopia [24] indicating that while Ghana has made significant strides, there are still opportunities for improvement in ensuring consistent use among all caregivers. The result presented in Table 6 provides a nuanced view of ITN ownership among caregivers with children under five years. Although the precise prevalence rate of ITN ownership was not detailed, the analysis indicates varying levels of ITN utilization among different demographic groups. Ethnic disparities are evident, with caregivers from the Ga/Dangme ethnic group showing significantly lower odds of high ITN utilization compared to those from the Akan ethnicity (aOR = 0.37, 95% CI = 0.15–0.92, p = 0.033). This discrepancy suggests that while ITN ownership might be similar across ethnic groups, utilization practices vary and could be influenced by cultural factors or differences in health education.

This current study shows that 97.3% of caregivers have heard of malaria, with 91.5% correctly identifying the bite of an infected mosquito as the cause. Despite this high awareness, 55.1% have not heard of insecticide-treated nets (ITNs). Among those familiar with ITNs, 51% strongly agree, and 46.5% agree that the primary purpose of using ITNs in children under five is to prevent malaria, with similar agreement levels on the need to use ITNs every night. However, 65% disagree that ITNs should be replaced or retreated every two years or after 20 washes, with most information about ITN usage coming from health facilities (60%). The knowledge of ITN usage among caregivers is reflected in the analysis of educational attainment. Contrary to expectations, caregivers with tertiary education were found to have lower odds of high ITN utilization compared to those with junior high school/middle school education (aOR = 0.31, 95% CI = 0.11–0.92, p = 0.035). This result suggests that higher educational levels do not necessarily correlate with better ITN usage. It may indicate that caregivers with more education might possess alternative knowledge or possibly have different perceptions about ITNs. Further research is needed to explore whether this finding is due to varying beliefs about malaria prevention or other health-related misconceptions among higher-educated caregivers.

Barriers associated with the utilization of insecticide-treated nets (ITNs) are factors that prevent or hinder the use of ITNs, despite their effectiveness in preventing malaria transmission. Several barriers to ITN utilization were identified in the study. Table 5 highlights significant associations between various socio-demographic factors, barriers, and knowledge of ITN utilization using Chi-square analysis. Factors such as sex (p = 0.025), educational level (p = 0.043), marital status (p < 0.001), religion (p = 0.020), challenges faced in using ITNs (p = 0.012), affordability (p = 0.001), and cultural beliefs (p = 0.003) all have significant associations with ITN knowledge. Another barrier is the availability of ITNs. In some areas, ITNs may not be readily available, either because they are not distributed through mass campaigns or because they are not stocked in local markets. The primary barrier identified in this study for non-ownership was being absent during distribution, which aligns with findings from Nigeria [25] where similar barriers were noted. In contrast, a study in Sierra Leone indicated that financial constraints and shortages were significant barriers to both ownership and usage [26]. Addressing these barriers through targeted outreach and education could enhance both ownership and utilization rates.

Similarly, a study in the Upper East Region found that some respondents did not have access to ITNs due to long distances to health facilities [27]. Low availability of ITNs may lead to low demand and poor utilization. A study conducted in Uganda found that the availability of ITNs was a major barrier to their use, with many households reporting that they could not find ITNs in local markets [28]. In some areas, ITNs may not be effective due to environmental factors, such as high humidity or frequent rainfall, which can cause the netting to become wet and lose its insecticidal properties. Caregivers who face challenges in consistently using ITNs are significantly less likely to utilize them (aOR = 0.50, 95% CI = 0.26–0.96, p = 0.040). Additionally, the perception of ITNs being “very affordable” is paradoxically associated with lower utilization (cOR = 0.45, 95% CI = 0.25–0.83, p = 0.011), though this association is not significant when adjusted for other factors (aOR = 0.63, 95% CI = 0.31–1.26, p = 0.198). This suggests that affordability alone may not be a sufficient determinant of ITN use, highlighting the need for comprehensive strategies that consider other socio-economic and cultural factors.

The finding that challenges in consistent use significantly reduce ITN utilization aligns with research conducted in Guinea, which identified similar barriers related to household size and access [20]. In that study, individuals living in larger households had lower usage rates, suggesting that practical barriers related to space and accessibility also play a critical role in ITN utilization. The paradoxical association between the perception of affordability and lower utilization is particularly interesting and resonates with findings from Nigeria, where increased access to ITNs did not translate into higher utilization rates [29]. This suggests that simply providing free or subsidized nets may not be enough; understanding the context in which these nets are perceived as affordable is crucial. The current study highlights the importance of socio-economic factors in determining ITN usage patterns. A study conducted in Mozambique indicated that poorer households had higher odds of sleeping under an ITN compared to wealthier households [30]. This contrasts with the findings of the current study, where perceptions of affordability did not lead to increased usage, emphasizing that socio-economic status must be considered holistically.

In line with the current findings, a study in Myanmar found a strong correlation between ownership and use of ITNs across different regions [31] However, it also noted that demographic factors such as age and marital status significantly influenced usage rates. The current study’s results suggest that addressing demographic disparities could enhance targeted interventions. Cultural perceptions regarding malaria prevention methods can also impact ITN utilization. Research in Nigeria indicated that cultural beliefs and practices significantly influenced whether individuals used available ITNs [32] Similarly, the current study indicates that perceptions around affordability might reflect deeper cultural attitudes towards health interventions. A study conducted in Nigeria found that many households did not sleep under ITNs every night, which reduced their effectiveness in preventing malaria transmission [29]. In some cultures, ITNs may be perceived as uncomfortable or unclean. A study conducted in Nigeria found that some households believed that ITNs were too hot to sleep under or that they could cause skin irritation [33]. The study also found that many households did not understand how to use ITNs correctly, leading to low utilization rates [33].

## Strength and limitation

The study demonstrated strengths, including a high response rate of 211 respondents, which enhances the reliability of findings on ITN ownership and utilization among caregivers with children under five. It utilized comprehensive data collection through structured questionnaires, providing detailed insights into socio-demographic factors, knowledge of malaria, and barriers to ITN use. However, the cross-sectional design limited the ability to infer causality and observe changes over time, while reliance on self-reported data may introduce bias. Additionally, findings may not be generalizable beyond Battor District due to differing socio-economic conditions and cultural beliefs in other regions.

## Conclusion

While ITN ownership and knowledge levels are relatively high among caregivers with children under five, gaps in utilization persist. The Ghana Health Service should improve distribution strategies, implement financial support programs, and conduct community education campaigns to promote the consistent use of ITNs. These efforts are essential for reducing malaria incidence and improving health outcomes for vulnerable populations in the Battor District. Addressing these factors is crucial for advancing toward the SDG 3.4 goal of enhancing mental health and well-being.

## Supporting information

S1 Text Questionnaire. (DOCX)
