# Supplementary material for: Insecticide-treated bed nets (ITN) ownership and utilization patterns among caregivers with children under five years: A community-based cross-sectional study in Battor, North Tongu District, Ghana
Source: PLOS Glob Public Health. 2025 Feb 6;5(2):e0004228. doi: 10.1371/journal.pgph.0004228 (PMC11801732; doi:10.1371/journal.pgph.0004228)
Supplement: S1 Text — (DOCX) [file pgph.0004228.s001.docx]

THE UNIVERSITY OF HEALTH AND ALLIED SCIENCES

FRED BINKA SCHOOL OF PUBLIC HEALTH, HOHOE

TOPIC**: Insecticide-Treated Bed Nets (ITN) Ownership and Utilization Patterns among Caregivers with Children Under Five Years in Selected District in Ghana”**

Dear Participants,

This research is being conducted by a student from the University of Health and Allied Sciences, School of Public Health- Hohoe. The purpose of this study is to examine Insecticide-Treated Bed Nets (ITN) Ownership and Utilization Patterns among Caregivers with Children under Five Years in Selected District in Ghana”. I therefore, encourage you to kindly respond to the questions with appropriate and valid answers to ensure accuracy and correct representation. No one will ask you to write your name on any part of the questionnaire. Data analysis will group all participants to protect anonymity. Your confidentiality and anonymity are assured in this study.

Please if you agree to participate, please answer the questions on this survey.

Thank you

**SECTION A: SOCIO-DEMOGRAPHIC FACTORS**

| No | QUESTION | RESPONSE (write or tick as required) |
| --- | --- | --- |
| A1 | What is your Age? | ……………….. |
| A2 | What is your Sex? | Male [ ]  Female [ ] |
| A3 | What is your Religion? | Christianity [ ]  Islam [ ]  African Traditional [ ]  Other specify …………………… |
| A4 | What Ethnicity do you belong to? | Akan [ ]  Ewe [ ]  Ga /Dangme [ ]  Guan [ ]  Mole/Dagbani [ ]  Other (specify)……………………. |
| A5 | What is your level of Educational? | No formal education [ ]  Primary [ ]  JHS/ Middle school [ ]  SHS/SSS/O Level/A Level [ ]  Tertiary [ ] |
| A6 | What is your Marital status? | Never married [ ]  Married [ ]  Divorced/Separated [ ]  Widowed [ ] |
| A7 | What is your employment status? | Civil/ Public servant [ ]  Artisan [ ]  Retiree [ ]  Unemployed [ ] |
| A8 | What is your household size | ………….. |
| **PREVALENCE OF INSECTICIDE-TREATED NET (ITN) OWNERSHIP AMONG CAREGIVERS** | | |
| B1 | Do you currently own insecticide-treated nets (ITNs) for malaria prevention purposes? | Yes [ ]  No [ ] |
| B2 | If No; what is the reason for not owning insecticide-treated nets (ITNS) | Not around during distribution [ ]  No child under five [ ]  Shortage of ITNs [ ]  No mosquito at my residence [ ]  Other Specify……. |
| B3 | How many ITNs do you currently possess for your household? | One [ ]  Two [ ]  Three and above [ ]  None [ ] |
| B4 | Where did you acquire the insecticide-treated nets (ITNs) | Community programme [ ]  Health facility[ ]  Pharmacy Shop [ ]  Friend/Relative [ ] |
| B5 | Did you and your child under five slept in ITN the night before this survey? | Yes [ ]  No [ ] |
| B6 | How beneficial is sleeping in ITNs with your household | Very beneficial [ ]  Benificial [ ]  Somehow beneficial [ ]  Not beneficial [ ] |
| **KNOWLEDGE OF ITN USAGE AMONG CAREGIVERS WITH CHILDREN UNDER FIVE:** | | |
| C1 | Have you heard of malaria? | Yes [ ]  No [ ] |
| C2 | What Causes Malaria? | Walking in the sun [ ]  Tiredness [ ]  Bite of Infected Mosquito [ ]  Not eating well [ ]  Don’t Know [ ] |
| C3 | Have you heard of insecticide-treated nets (ITNs)? | Yes [ ]  No [ ] |
| C4 | The primary purpose of using insecticide-treated nets (ITNs) in children under five is to prevent malaria? | Strongly agree [ ]  Agree [ ]  Disagree [ ]  Strongly disagree [ ] |
| C5 | ITNs should be used every night to effectively prevent malaria in children under five? | Strongly agree [ ]  Agree [ ]  Disagree [ ]  Strongly disagree [ ] |
| C6 | Every 2 years or after 20 washing, ITNs should be replaced or retreated with insecticide for effective malaria prevention? | Strongly agree [ ]  Agree [ ]  Disagree [ ]  Strongly disagree [ ] |
| C7 | Where did you acquire information about ITN usage for children under five? | Health facilities [ ]  Community programs [ ]  Media (TV, radio, etc.) [ ]  Family/friends [ ] |
| **BARRIERS ASSOCIATED WITH THE UTILIZATION OF INSECTICIDE-TREATED NETS AMONG CAREGIVERS WITH CHILDREN UNDER FIVE YEARS** | | |
| D1 | Have you faced challenges in consistently using ITNs for your children under five? | Yes [ ]  No [ ] |
| D2 | If yes, please specify the main challenges faced in using ITNs regularly | Cost [ ]  Availability [ ]  Allergic to chemical [ ]  Inconvenience/ heat [ ]  Shape texture [ ] |
| D3 | How affordable are ITNs for your household? | Very affordable [ ]  Affordable [ ]  Moderately affordable [ ]  Not affordable [ ] |
| D4 | Do cultural beliefs significantly affect your decision or ability to use ITNs for your children under five? | Strongly agree [ ]  Agree [ ]  Disagree [ ]  Strongly disagree [ ] |
